# Oscillating magnetic field disrupts magnetic orientation in Zebra finches, *Taeniopygia guttata*

**DOI:** 10.1186/1742-9994-6-25

**Published:** 2009-10-23

**Authors:** Nina Keary, Tim Ruploh, Joe Voss, Peter Thalau, Roswitha Wiltschko, Wolfgang Wiltschko, Hans-Joachim Bischof

**Affiliations:** 1 Lehrstuhl Verhaltensforschung, Universität Bielefeld, Postfach 100131, D-33501 Bielefeld, Germany; 2 FB Biowissenschaften, J. W. Goethe-Universität, Siesmayerstr. 70, D-60054 Frankfurt/Main, Germany

## Abstract

**Background:**

Zebra finches can be trained to use the geomagnetic field as a directional cue for short distance orientation. The physical mechanisms underlying the primary processes of magnetoreception are, however, largely unknown. Two hypotheses of how birds perceive magnetic information are mainly discussed, one dealing with modulation of radical pair processes in retinal structures, the other assuming that iron deposits in the upper beak of the birds are involved. Oscillating magnetic fields in the MHz range disturb radical pair mechanisms but do not affect magnetic particles. Thus, application of such oscillating fields in behavioral experiments can be used as a diagnostic tool to decide between the two alternatives.

**Methods:**

In a setup that eliminates all directional cues except the geomagnetic field zebra finches were trained to search for food in the magnetic north/south axis. The birds were then tested for orientation performance in two magnetic conditions. In condition 1 the horizontal component of the geomagnetic field was shifted by 90 degrees using a helmholtz coil. In condition 2 a high frequently oscillating field (1.156 MHz) was applied in addition to the shifted field. Another group of birds was trained to solve the orientation task, but with visual landmarks as directional cue. The birds were then tested for their orientation performance in the same magnetic conditions as applied for the first experiment.

**Results:**

The zebra finches could be trained successfully to orient in the geomagnetic field for food search in the north/south axis. They were also well oriented in test condition 1, with the magnetic field shifted horizontally by 90 degrees. In contrast, when the oscillating field was added, the directional choices during food search were randomly distributed. Birds that were trained to visually guided orientation showed no difference of orientation performance in the two magnetic conditions.

**Conclusion:**

The results indicate that zebra finches use a receptor that bases on radical pair processes for sensing the direction of the earth magnetic field in this short distance orientation behavior.

## Background

The use of the earth magnetic field for spatial orientation has been shown for various animals over the last years. Among the vertebrates, a magnetic compass has been demonstrated in fish [1,2], amphibians [3], reptiles [4] and mammals [5-7]. Birds, however, is by far the best studied group where magnetic orientation is concerned (for review, see [8]). Migratory birds use the magnetic compass sense for finding the migratory direction [9]. Non migratory birds like pigeons (*Columba livia *f. *domestica*) use it to find the direction towards their home loft [10], and domestic chickens (*Gallus gallus*) [11,12] and zebra finches (*Taeniopygia guttata*) [13] can be trained to use the geomagnetic field for orientation over short distances. However, the physiological mechanisms necessary to perceive the magnetic field information still remain largely unexplained. Two hypotheses are discussed. One hypothesis is based on the demonstration of clustered iron-rich deposits in the ethmoid region and upper beak of the birds, consisting of magnetite and maghemite [14-16]. Depending on the strength and the direction of the geomagnetic field, such deposits may be affected and exert mechanical forces onto adjacent cell membranes [17,18]. By alteration of the membrane conductivity for ions, the magnetic field information could then be translated into a neuronal signal, which may be conveyed via the ophthalmic branch of the trigeminal nerve [19].

The second hypothesis is based on interactions of the geomagnetic field with specialized photoreceptor molecules in the retina of the birds [20,21]. Light-induced electron transfer creates a radical pair with two possible spin states. The rate and the products of this reaction are affected by the dynamics of the transition between the two states, which in turn depends on the alignment of the molecule in the geomagnetic field. Assuming that the molecules are aligned radially within the retina of the half-sphere shaped eyeball, each receptor molecule at different retina sites has a different angle to the geomagnetic field, and will thus be modulated differently. According to Schulten and Windemuth [20] and Ritz [21], this modulation may result in a "pattern" superimposed on the visual image, which allows the birds to derive information on magnetic field orientation by appropriate (as yet unknown) neuronal mechanisms.

One prediction of the radical pair hypothesis is that oscillating magnetic fields in the MHz range induce resonance effects on radical-pair spin states, thus disrupting the magnetosensitive function of the receptor molecules. One of the effective frequencies is the "Larmor frequency". It is the frequency of spin precession which depends on the intensity of the ambient magnetic field. Migratory robins [22,23] and directionally trained chickens [12] were disoriented when such a high frequency magnetic field was added to the local geomagnetic field oscillating at an angle to the magnetic vector. The iron-based receptor mechanism in the beak, however, would not be affected by these oscillating fields due to its physical properties. Thus, the technique can be applied as a diagnostic tool to decide whether magnetic field perception is based on radical pair processes [24]. It was, besides the above mentioned examples in birds, already helpful to investigate the physiological basis of magnetic field perception in mammals. The magnetic field orientation of mole-rats (Cryptomys anselli) was not disturbed by application of oscillating magnetic fields [25]; their magnetic sense thus seems to be based on a different physical principle, probably involving magnetite [26].

In the present study, we trained zebra finches to search for food in the north/south axial direction with the geomagnetic field as the only orientation cue. After the training we examined the orientation performance of the birds in a magnetic field with North shifted by 90° and in a situation where the static field was superimposed by a weak magnetic field oscillating with the Larmor frequency. To test for possible unspecific side effects of the oscillating field on other physiological mechanisms, not associated with the magnetosensory system, we trained a second group of birds to search for food in a spatial memory task based on visual landmarks and tested their orientation performance in the same two magnetic conditions.

## Methods

17 zebra finches of both sexes from the institute's breeding colony were used for the study, ten birds (5 male/5 female) were used for the magnetic orientation experiment, seven (4 male/3 female) were trained in the spatial orientation task based on visual perception.

**Figure 1:**
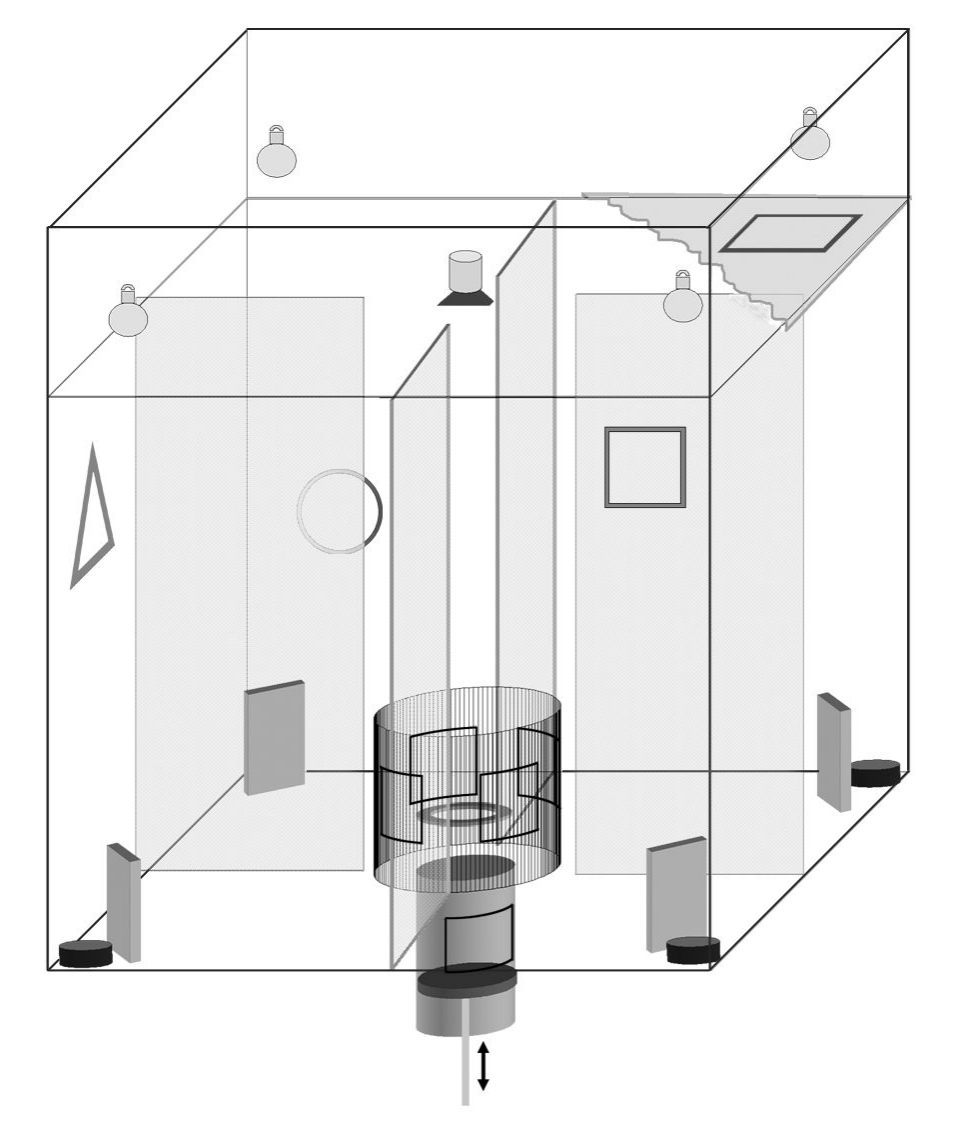
**Experimental setup**. Experimental setup used for training and testing the birds. The whole box could be rotated prior to each experimental trial. Visual landmarks (squares, circles and triangles) were only applied for the visual orientation task. The Helmholz coil for shifting the geomagnetic field is not shown in the picture. For details see methods.

The experiments for both, magnetic and visual orientation were performed in a cubic box (80 × 80 × 80 cm) of non-magnetic plastic material (Fig. 1). The ceiling consisted of opaque plastic with a small circular hole in the center, bearing the front lens of a miniature video camera. A circular cage, with a circular perch inside and a door to each of the four side compartments, was positioned in the center. An elevator system was installed in the middle of the cage floor which allowed lifting the birds into the center cage from below without giving them a directional bias. The four doors of the center cage could be opened simultaneously from the outside by the experimenter.

In the magnetic orientation experiment the setup was homogeneously illuminated and rotated by 90° to a new position prior to each experimental trial. The partitions between the compartments were randomly exchanged to keep the birds from visually guided orientation. In each corner a food tray was placed behind a screen, so that it was not visible from the center of the box. In this experiment food was available at two trays in north-south direction, in east-west direction the food trays were covered with transparent mesh tissue so that olfactory cues were still available.

For the second experiment, testing visually guided orientation, the setup was slightly changed. Visual landmarks were positioned outside the center cage, at the inner walls of the setup. The landmarks were squares, circles and triangles made of blue, green and red paper with 10 cm diameter. The spatial relation of all landmarks inside the box remained unchanged and could be used for orientation, whereas the whole setup was rotated prior to each trial, to make sure that the correct food tray was located in different magnetic directions.

Before the training session, the zebra finches were deprived of food and water overnight. At the beginning of each trial, a bird was kept in a circular cage in the center of the box for two minutes to adapt to the experimental situation. Then, the four doors to the side compartments were opened simultaneously and the bird was allowed to explore the whole environment and to search for food. For training the magnetic orientation task the zebra finches were exposed to the local magnetic field of the earth (intensity: 43 μT, inclination: 67°). Previous experiments had shown that the zebra finches chose an axis rather than a unimodal direction [13], therefore the birds were rewarded "axially" by access to food in the hidden trays at both ends of the north-south axis. In addition to the food reward at the correct directions, the birds were "punished" by a 25 s darkness period and were not allowed any longer to search for food in that trial if they chose a wrong direction. For visually guided spatial orientation, the birds were trained using the same procedure. In this case food was available in only one direction.

The behavior of the birds was monitored using the small CCD-camera on the ceiling of the setup. For each bird, we counted the direction of the first attended tray as directional decision, if the bird came close enough to be able to look behind the screen and to examine the tray. The zebra finches were trained at least 3 times a day in random order until they had learned the correct magnetic axis or the correct direction in the spatial memory task. Seven correct choices out of eight in a row were defined as the criterion for successfully learning the magnetic axis (binominal test, p < 0.05). To make the data comparable, the same criterion was applied to the visual orientation task.

The birds that passed the criterion for learning the magnetic axis were then tested for magnetic orientation in two conditions. Each bird was tested ten times in each of two conditions, the first inspection of the food tray was taken as directional choice. Both conditions were applied in a pseudo random sequence, first, to avoid long term behavioral/experimental effects to influence data acquisition, and second, to keep the birds motivated by reinforcement in one of the two conditions, where correct orientation behavior was expected.

In condition 1, the horizontal component of the earth magnetic field was experimentally shifted clockwise by 90° using a Helmholtz coil with 2 m diameter, with intensity and inclination not affected. For condition 2, an oscillating field with the local Larmor frequency of 1.156 MHz and an amplitude of 0.47 μT (about 1% of the intensity of the local geomagnetic field) was added to the 90° shifted field, using a coil antenna around the test arena. The coil antenna was mounted horizontally to apply an HF field oscillating at an angle of 23° to the magnetic vector. Oscillating currents from a high frequency (HF) generator were amplified by a HF amplifier and fed into the coil through an upstream resistance of 51 Ù. The coil consisted of a double winding of coaxial cable with 2 cm of the screening removed opposite the feed. For the condition with the static field only, the HF field was switched off by unplugging the output connector of the HF amplifier. The oscillating field and the geomagnetic field were measured daily preceding each experimental session, using a spectrum analyser (Hewlett Packard) and a high resolution fluxgate sensor (Stefan Mayer Instruments).

All birds in the second experiment had learned the visually guided spatial orientation task in the geomagnetic field and they were also tested under the two magnetic conditions in pseudo random order. In condition 1, the visual orientation performance of the birds was tested in a magnetic field shifted clockwise by 90°. In condition 2, the birds had to perform the task in the same static magnetic field with additional application of the same oscillating magnetic field as used in the magnetic field orientation experiment (1.156 MHz/0.47 μT amplitude).

The data were statistically analyzed by calculating scores of correct and incorrect choices for each bird and for both test conditions (Formula: number of correct choices/number of all choices). The higher the score, the more directional choices were in the correct axis (magnetic orientation) or direction (visual orientation).

Mean data for each test condition were tested against random distribution of 0.5 (axial magnetic orientation) or 0.25 (visual orientation) with a one sample t-test. For each experiment the mean differences between both test conditions were statistically evaluated using the Wilcoxon test.

The original research reported herein was performed in accordance with the guidelines and laws for Animal Welfare in Germany.

## Results

### Magnetic orientation

Seven (4 male/3 female) of ten zebra finches reached the criterion that we defined for learning to orient after the geomagnetic field. The birds needed different numbers of training trials, ranging between 7 and 16 trials for learning. These birds were then tested ten times for their orientation performance in a magnetic field with magnetic North shifted by 90° alone, and ten times in the same field with a vertically oscillating field of 1.156 MHz superimposed; both conditions in pseudo-random order. The respective scores are given in Table 1.

**Table 1:** Orientation performance scores of all birds tested in the magnetic orientation task and in the visually guided orientation task

**magnetic orientation**			**visual orientation**		
**bird**	**shifted field**	**shifted field** **+** **HF**	**bird**	**shifted field**	**shifted field** **+** **HF**

b1	0.7	0.4	b11	0.9	1

b2	0.9	0.5	b12	0.6	0.7

b3	0.7	0.4	b13	1	1

b4	0.7	0.3	b14	1	0.7

b5	0.6	0.4	b15	0.9	1

b6	0.8	0.8	b16	0.9	0.8

b7	0.8	0.6	b17	1	0.9

**mean**	**0.74**	**0.49**	**mean**	**0.9**	**0.87**

**SD**	**0.098**	**0.17**	**SD**	**0.14**	**0.14**

Scores were calculated from the number of correct and incorrect choices of each bird. A score of 1 shows that all choices were in the correct axis (for magnetic orientation) or in the correct direction (for visual orientation), 0 determined no correct choice.

**Figure 2:**
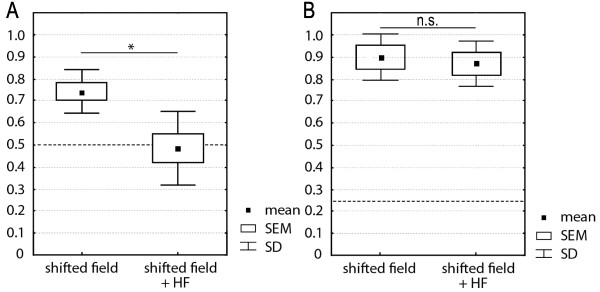
**Mean orientation scores after magnetic (A) and visually guided (B) orientation**. Both experiments were performed in two magnetic conditions, a 90° shifted static magnetic field and in a shifted field with an additionally applied weak HF field. Reference values depicted as dashed lines: 0.5 for magnetic (axial) orientation, 0.25 for visual (directional) orientation. For details see text.

Statistical analysis revealed that with a mean score of 0.74 (± 0.098 SD) the birds were significantly oriented in the 90° horizontally shifted field (n = 7, t = 6.58, p = 0.0006) (fig. 2).

If the same birds were tested in the shifted field plus the oscillating field, they did not show any orientation, reaching orientation scores between 0.4 and 0.6 (Table 1). With scores of 0.8, one bird, however, was oriented in both test conditions. The t-test over all birds (n = 7) revealed that the mean score of 0.49 (± 0.17 SD) with the oscillating field added did not differ from random (reference value = 0.5, n = 7, t = -0.23, p = 0.83) (fig. 2A).

To test for differences of the orientation performance in both conditions, the mean scores were statistically analyzed, showing that there is a significant difference of the orientation performance in the shifted field compared with that shown in a field with an additionally applied HF field (n = 7, Z = 2.2, p = 0.028).

### Visual orientation

The second group of birds was trained in the geomagnetic field to visually guided spatial orientation and then likewise tested in both, the 90° horizontally shifted magnetic field, and in a shifted field with an additionally applied HF component. With mean orientation scores of 0.9 ± 0.14 SD (shifted magnetic field) and 0.87 ± 0.14 SD (shifted magnetic field + HF) the data clearly show that the birds are well oriented in both conditions (fig. 2B), as both mean scores differ significantly from chance level (reference level = 0.25, n = 7, t = 12.2, p = 0.00002 for visual orientation in the shifted magnetic field/n = 7, t = 11.9, p = 0.00002 for orientation in the HF condition). Statistical analysis reveals no differences in the orientation performance in both conditions (n = 7, Z = 0.31, p = 0.75). Statistical analysis using a t-test shows that visually guided orientation performance in the shifted magnetic field is significantly better compared to orientation after the shifted magnetic field in the first experiment (n = 7, t = 2.42, p = 0.39).

## Discussion

The first of the experiments confirms our previous demonstration of the ability of zebra finches to orient after the earth magnetic field [13]. Shifting the horizontal component of the geomagnetic field by 90° does not lead to a disruption of orientation; rather the birds use the respective shifted direction for food search. Our present experiments additionally show that the birds are no longer oriented in a shifted geomagnetic field superimposed by a weak magnetic field oscillating with the Larmor frequency. In contrast, the superimposed oscillating magnetic field had no effect on visually guided spatial orientation.

These results clearly indicate that the oscillating magnetic field does not have any effect on the general orientation performance of the birds. Its effect is only visible if the birds have to use the magnetic field for orientation. According to the theories outlined above, such an effect can only be expected if the perception mechanism involves radical pair processes. Thus zebra finches do not use the "beak receptor" consisting of iron rich deposits around the beak because these, according to Ritz et al. [24], can not be affected by high frequency magnetic fields due to its physical properties. Instead, such an effect can be expected if, according to the hypothesis of Schulten and Windemuth [20] and Ritz et al. [21], the direction of the geomagnetic field is mediated by radical pair processes in specialized photopigments in the eye.

Cryptochrome, a photopigment with a flavin as photoactive chromophore [27], has been suggested by Ritz et al. [21] as a candidate. It was first described in plants, and later on found in the retina of chickens [28,29] and in three species of passerine birds [30,31], among them the zebra finch [31]. Although there is as yet no direct proof of a relation of cryptochrome with the radical pair process, it is remarkable that this pigment is found in the retina of all birds which have been shown to be affected by oscillating magnetic fields in magnetic orientation.

In the first experiment, testing for magnetic orientation, one bird was oriented in the 90° shifted field as well as in the magnetic field which was superimposed by the oscillating field. It is not to assume that this single bird was still able to orient with the help of the static magnetic field in the HF condition. Although we took meticulous care with the prevention of any other orientational cue, it might have found a way to orient without the use of the geomagnetic field. More plausible, however, is the assumption that it reached the high score just by chance.

The second part of our experiment was designed because an oscillating magnetic field, besides its effect on magnetic field receptors, could have more general effects on other physiological mechanisms, leading to a general disorientation of the animals irrespective of the sensory cues. If there were such general effects, other spatial orientation tasks should likewise be affected by oscillating magnetic fields. As stated above, magnetoreception may be an additional "specialized" function of the visual system. Indeed, modulation of the visual percept by changing the direction and strength of magnetic fields has been shown in humans [32]. Thus, to look for side effects generated by the HF field, it was most plausible to do this with a task based on visual perception. The present results clearly demonstrate that application of HF fields does not affect visual perception or other physiological mechanisms used for spatial orientation, as the birds were well oriented in all magnetic conditions during the visual orientation task. As stated above, oscillating magnetic fields thus have an effect specifically on magnetic orientation, indicating that radical pair processes are involved in perception.

Our data show that orientation performance with the use of visual landmarks is more effective compared to that with magnetic cues. Our experiments support previous ones [33] which have already shown that zebra finches perform very well in spatial orientation tasks where food had to be found with help of distant visual landmarks. Food search may therefore mainly be based on visual cues. It may well be that the use of the different perception systems is dependent on the distance between the bird and the food to be found. In a situation where the food is in the near vicinity (a radius of centimeters to meters), the use of a compass mechanism might not be the usual solution. If the bird has to travel hundreds or thousands of meters between nest- and feeding site the use of a compass sense might be helpful.

Because the distance from the starting point of the bird to the food tray in our experiments was under 100 cm, this might explain the obtained difference in orientation performance based on magnetic and visual information, respectively.

The birds in our experiments were probably forced to use the magnetic information, which would never have been used under normal conditions for food search over such short distances.

Our experiments add another example to the list of species which are using radical pair mechanisms for the perception of magnetic field parameters. Besides migratory birds like the robin, it has been shown for chickens which can use the magnetic sense for orientation within distances of less than one meter. Domestic chickens have a comparably small home range and do not accomplish long distances. Zebra finches, in contrast, are also not migratory, but they do ramble around over extended distances. Thus the use of the radical pair mechanisms may not be related to the lifestyle of an animal.

Because it has been shown in two passerine species as well as in a galliform species, two evolutionary branches of birds that already separated early in the late cretaceous more than 90 millions years ago [34], this perceptual mechanism might be a very old one and may have already existed in their common ancestor. There is, however, first evidence that the mechanism is not to be found in all vertebrates. As stated above, in some of the mammalian species investigated so far, magnetic perception seems to be based on the alternative magnetite based receptor [25].

## Conclusion

Our results unequivocally show that magnetic field orientation in the zebra finch is disturbed by superimposing high frequency oscillating magnetic fields. According to theory [20,21], this indicates that the perception of the earth magnetic field in zebra finches is based on a molecular radical pair process. As the radical pair based compass sense has also been demonstrated in other birds from two evolutionary branches [12,22,23] that separated already early in evolution, this perceptual mechanism is obviously quite common among birds.

## Competing interests

The authors declare that they have no competing interests.

## Authors' contributions

NK made substantial contributions to conception and design of the experiment and acquisition of data. TR contributed to the setup conception, acquired data and performed part of the statistical analysis. JV designed the experiment, supervised the data acquisition and analysis and drafted the manuscript. PT built up and maintained the technical facilities for HF stimulation and was involved in drafting the manuscript. RW was involved in drafting the manuscript and revising it critically for important intellectual content. WW contributed to the setup design and revised the manuscript for important intellectual content. HB made substantial contributions to experimental concept, drafting the manuscript and statistical analysis of data.

## References

[B1] Quinn TP (1980). Evidence for celestial and magnetic compass orienttaion in lake migrating sockeye salmon fry. J comp Physiol.

[B2] Taylor PB (1986). Experimental evidence for geomagnetic orientation in juvenile chinook salmon, Oncorhynchus tschawytscha. J Fish Biol.

[B3] Philipps JB (1986). Magnetic compass orientation in the eastern red.spotted newt (Notophtalmus viridescens). J comp Physiol A.

[B4] Lohmann KJ (1991). Magnetic orientation by hatchling loggerhead sea turtles (Caretta caretta). J Exp Biol.

[B5] Burda H, Marhold S, Westenberger T, Wiltschko R, Wiltschko W (1990). Magnetic compass orientation in the subterranean rodent Cryptomys hottentotus (Bathyergidae). Experientia.

[B6] Wang Y, Pan Y, Parsons S, Walker M, Zhang S (2007). Bats respond to polarity of a magnetic field. Proc Biol Sci.

[B7] Muheim R, Edgar NM, Sloan KA, Phillips JB (2006). Magnetic compass orientation in C57BL/6J mice. Learn Behav.

[B8] Wiltschko W, Wiltschko R (2007). Magnetoreception in birds: two receptors for two different tasks. J Ornithol.

[B9] Wiltschko W (1968). On the effect of static magnetic fields on the migratory orientation of the robin (Erithacus rubecula). Z Tierpsychol.

[B10] Walcott C, Green RP (1974). Orientation of homing pigeons altered by a change in the direction of an applied magnetic field. Science.

[B11] Freire R, Munro UH, Rogers LJ, Wiltschko R, Wiltschko W (2005). Chickens orient using a magnetic compass. Curr Biol.

[B12] Wiltschko W, Freire R, Munro U, Ritz T, Rogers L, Thalau P, Wiltschko R (2007). The magnetic compass of domestic chickens, Gallus gallus. J Exp Biol.

[B13] Voss J, Keary N, Bischof HJ (2007). The use of the geomagnetic field for short distance orientation in zebra finches. Neuroreport.

[B14] Beason RC, Nichols JE (1984). Magnetic orientation and magnetically sensitive material in a transequatorial migratory bird. Nature.

[B15] Williams MN, Wild JM (2001). Trigeminally innervated iron-containing structures in the beak of homing pigeons, and other birds. Brain Res.

[B16] Fleissner G, Holtkamp-Rotzler E, Hanzlik M, Winklhofer M, Fleissner G, Petersen N, Wiltschko W (2003). Ultrastructural analysis of a putative magnetoreceptor in the beak of homing pigeons. J Comp Neurol.

[B17] Kirschvink JL, Gould JL (1981). Biogenic magnetite as a basis for magnetic field detection in animals. Biosystems.

[B18] Fleissner G, Stahl B, Thalau P, Falkenberg G, Fleissner G (2007). A novel concept of Fe-mineral-based magnetoreception: histological and physicochemical data from the upper beak of homing pigeons. Naturwissenschaften.

[B19] Beason R, Semm P (1996). Does the avian ophthalmic nerve carry magnetic navigational information?. J Exp Biol.

[B20] Schulten K, Windemuth A, Maret G, Boccara N, J K (1986). Model for a physiological magnetic compass. Biophysical Effects of Steady Magnetic Fields.

[B21] Ritz T, Adem S, Schulten K (2000). A model for photoreceptor-based magnetoreception in birds. Biophys J.

[B22] Thalau P, Ritz T, Stapput K, Wiltschko R, Wiltschko W (2005). Magnetic compass orientation of migratory birds in the presence of a 1.315 MHz oscillating field. Naturwissenschaften.

[B23] Ritz T, Wiltschko R, Hore PJ, Rodgers CT, Stapput K, Thalau P, Timmel CR, Wiltschko W (2009). Magnetic compass of birds is based on a molecule with optimal directional sensitivity. Biophys J.

[B24] Ritz T, Thalau P, Phillips JB, Wiltschko R, Wiltschko W (2004). Resonance effects indicate a radical-pair mechanism for avian magnetic compass. Nature.

[B25] Thalau P, Ritz T, Burda H, Wegner RE, Wiltschko R (2006). The magnetic compass mechanisms of birds and rodents are based on different physical principles. J R Soc Interface.

[B26] Wegner RE, Begall S, Burda H (2006). Magnetic compass in the cornea: local anaesthesia impairs orientation in a mammal. J Exp Biol.

[B27] Sancar A (2003). Structure and function of DNA photolyase and cryptochrome blue-light photoreceptors. Chem Rev.

[B28] Bailey MJ, Chong NW, Xiong J, Cassone VM (2002). Chickens' Cry2: molecular analysis of an avian cryptochrome in retinal and pineal photoreceptors. FEBS Lett.

[B29] Haque R, Chaurasia SS, Wessel JH, Iuvone PM (2002). Dual regulation of cryptochrome 1 mRNA expression in chicken retina by light and circadian oscillators. Neuroreport.

[B30] Moller A, Sagasser S, Wiltschko W, Schierwater B (2004). Retinal cryptochrome in a migratory passerine bird: a possible transducer for the avian magnetic compass. Naturwissenschaften.

[B31] Mouritsen H, Janssen-Bienhold U, Liedvogel M, Feenders G, Stalleicken J, Dirks P, Weiler R (2004). Cryptochromes and neuronal-activity markers colocalize in the retina of migratory birds during magnetic orientation. Proc Natl Acad Sci USA.

[B32] Thoss F, Bartsch B (2003). The human visual threshold depends on direction and strength of a weak magnetic field. J Comp Physiol A Neuroethol Sens Neural Behav Physiol.

[B33] Watanabe S, Bischof HJ (2004). Effects of hippocampal lesions on acquisition and retention of spatial learning in zebra finches. Behav Brain Res.

[B34] Ericson PG, Anderson CL, Britton T, Elzanowski A, Johansson US, Kallersjo M, Ohlson JI, Parsons TJ, Zuccon D, Mayr G (2006). Diversification of Neoaves: integration of molecular sequence data and fossils. Biol Lett.

